# Evaluation of Molecular Methods to Identify Chagas Disease and Leishmaniasis in Blood Donation Candidates in Two Brazilian Centers

**DOI:** 10.3390/pathogens12040508

**Published:** 2023-03-24

**Authors:** Juliana de Jesus Guimarães Ferreira, Sandra Cecília Botelho Costa, Marcelo Addas-Carvalho, Mariane Barroso Pereira, Adriana de Oliveira França, Rodrigo Gonçalves de Lima, Paula Durante Andrade, Jamiro da Silva Wanderley, Luiz Cláudio Martins, Eros Antonio de Almeida, Gláucia Elisete Barbosa Marcon

**Affiliations:** 1 Laboratório de Diagnóstico de Doenças Infecciosas por Técnicas de Biologia Molecular, Departamento de Clínica Médica, Faculdade de Ciências Médicas, Universidade Estadual de Campinas (UNICAMP), Campinas 13083-887, SP, Brazil; 2 Centro de Hematologia e Hemoterapia, Universidade Estadual de Campinas (UNICAMP), Campinas 13083-887, SP, Brazil; 3 Laboratório de Doenças Infecciosas e Parasitárias (LabDIP), Programa de Pós-Graduação em Doenças Infecciosas e Parasitárias, Universidade Federal de Mato Grosso do Sul, Cidade Universitária s/n, Campo Grande 79090-900, MS, Brazil; 4 Grupo de Estudos em Doença de Chagas (GEDoCh), Departamento de Clínica Médica, Faculdade de Ciências Médicas, Universidade Estadual de Campinas (UNICAMP), Campinas 13083-887, SP, Brazil; 5 Fundação Oswaldo Cruz Mato Grosso do Sul (FIOCRUZ MS), Rua Gabriel Abrão, 92, Jardim das Nações, Campo Grande 79081-746, MS, Brazil

**Keywords:** Chagas disease, leishmaniasis, blood donation, melting curve analysis, PCR, nPCR, qPCR

## Abstract

In Brazil, blood donation is regulated by the Brazilian Ministry of Health, and all States follow the same protocol for clinical and laboratory screening. Brazil is an endemic country for Chagas disease (CD), caused by *Trypanosoma* cruzi, and for leishmaniasis, caused by a species of *Leishmania* spp. Screening for leishmaniosis is not routinely performed by blood banks. Given the antigenic similarity between *T. cruzi* and *Leishmania* spp., cross-reactions in serological tests can occur, and inconclusive results for CD have been found. The objective of this study was to apply molecular techniques, e.g., nPCR, PCR, and qPCR, to clarify cases of blood donation candidates with non-negative serology for CD and to analyze the difference between the melting temperature during real-time PCR using SYBR Green. Thirty-seven cases that showed non-negative results for CD using chemiluminescent microparticle immunoassay (CMIA) tests from blood banks in Campo Grande, MS, and Campinas, SP, were analyzed. In the serum samples, 35 samples were evaluated by ELISA, and 24.3% (9/35) showed positive results for CD. nPCR was able to detect 12 positive results in 35 samples (34.28%). qPCR for *T. cruzi* was quantifiable in the samples that showed a value ≥0.002 par eq/mL (parasite equivalents per milliliter), and in 35 samples, 11 (31.42%) were positive. Of all evaluated samples using the described tests (CMIA, ELISA, nPCR, and qPCR), 18 (48.6%) were positive for CD. For MCA by qPCR, the melting temperature was 82.06 °C ± 0.46 for *T. cruzi* and 81.9 °C ± 0.24 for *Leishmania infantum*. The Mann–Whitney test showed a significant value of *p* < 0.0001. However, the differentiation between *T. cruzi* and *L. infantum* could not be considered due to temperature overlap. For leishmaniasis, of the 35 samples with non-negative serology for CD tested by the indirect fluorescent antibody test (IFAT), only one sample (2.85%) was positive (1:80). The PCR for *Leishmania* spp. was performed on 36 blood samples from donation candidates, and all were negative. qPCR for *L. infantum* showed 37 negative results for the 37 analyzed samples. The data presented here show the importance of performing two different tests in CD screening at blood banks. Molecular tests should be used for confirmation, thereby improving the blood donation system.

## 1. Introduction

Blood donation is an important issue for preserving the lives of different subjects with compromised health issues. In Brazil, 4,724,288 people were eligible for blood donations in 2017. Of this number, 3,790,062 could actually donate blood according to Brazilian law [1]. One of the main reasons for deferring blood donations is the presence of microorganisms causing infectious disease, e.g., *Trypanosoma cruzi*, an etiologic agent of Chagas disease (CD) [2]. CD is distributed mainly in Latin America, affecting 6 to 7 million people worldwide [3]. Blood donations were the second main way of transmitting the parasite *T. cruzi*, with vectorial transmission being the first [4]. The inclusion of serological screening for Chagas disease at Brazilian blood banks has decreased transmission rates. However, population migrations from Latin America to other continents have made international rates increase, mainly in the USA and Spain [4,5]. Furthermore, recent studies have demonstrated the presence of *Leishmania* spp. DNA and anti-*Leishmania* antibodies in the blood of donation candidates, both in endemic and non-endemic countries [6,7,8,9]. 

Leishmaniasis is another disease that has spread to 98 countries around the world, affecting 1 million people per year [10]. There is a wide range of species of *Leishmania* that cause the disease, as well as a great variety of vectors that can transmit them, hampering proper treatments and controls. Although many subjects will develop a cutaneous or visceral form of the disease, according to the species responsible for the infection, there is a fraction of people that will not present any specific symptoms related to the disease [11,12].

Both parasites belong to the Kinetoplastidae order and Trypanosomatidae family, so they show many similar characteristics, such as morphology (i.e., cell structure and surface coat), life cycle, metacyclogenesis, and genetics. The most peculiar organelle of the parasites is the kinetoplast DNA (kDNA), where they have extra DNA organized in maxicircles and minicircles, the latter presenting repetitive sequences that seem to be responsible for its species-specific nature [13,14,15].

The aforementioned characteristics have important issues related to the diagnosis of diseases, because carriers may not manifest specific symptoms, and both diseases may be occurring simultaneously at the same place, since there is evidence of invertebrate and vertebrate hosts presenting mixed infections, hampering proper identification of the parasite. Some reports show cross-reactivity in serological tests aiming to identify specific antibodies against *T. cruzi* [16]. Although the World Health Organization (WHO) accounts for two different serological tests to confirm the chronic Chagas disease diagnosis, the assays employed depend on each center’s criteria, with the most commonly used tests being the enzyme-linked immunosorbent assay (ELISA), the indirect fluorescent antibody test (IFAT), indirect hemagglutination (IHA), and chemiluminescent microparticle immunoassays (CMIAs) [17,18].

According to a determination from the Ministry of Health of Brazil, the detection of anti-*T cruzi* antibody must be performed at blood banks using the ELISA or CMIA methods [18]. The use of recombinant antigens or chimeric proteins has been contributing to improved serological assays for Chagas disease identification or even for differentiation between Chagas disease and leishmaniasis in places where both diseases are co-endemic, minimizing the chances of cross-reactions [19,20,21,22,23]. In the case of inconclusive Chagas disease results, donated blood samples must be collected to carry out complementary laboratory investigations to confirm the initial result.

In the last ten years, studies have been carried out to improve methods for detecting *T. cruzi* DNA, which have been applied to inconclusive test results, for evaluating treatment efficacy, for quantitative analysis through quantitative PCR (qPCR), and for genotyping parasite discrete typing units (DTUs) [24,25]. On the other hand, the specific qPCR using the SYBR Green assay, which allows for melting curve analysis (MCA), has been used to identify the two different *Leishmania* subgenera or even differentiate some species within each *Leishmania (Leishmania)* or *Leishmania (Viannia)* subgenus, which is very important for identifying the correct treatment, for patient evaluations and follow-ups, for severe cases with drug resistance, and for epidemiological surveys [26,27,28,29,30,31,32,33].

Since leishmaniasis serology may indicate serological scars, it is necessary to improve the diagnosis tests that will clarify inconclusive serology cases for Chagas disease. This study applies the MCA methodology to differentiate *T. cruzi* parasites from the *Leishmania* genus in blood samples from donation candidates with non-negative (i.e., inconclusive or positive) serology tests for Chagas disease and to confirm parasite identification through polymerase chain reaction (PCR) and nested polymerase chain reaction (nPCR).

## 2. Methods

### 2.1. Participants

The 66 participants in this prospective study were as follows: (a) Twenty-eight (28) individuals from the Hematology and Hemotherapy Center at the Campinas State University (Hemocentro/Unicamp), Campinas, SP, Brazil; (b) nine (9) individuals from the “Jose Scaff Hematology and Hemotherapy Center” in Mato Grosso do Sul (Hemosul), Campo Grande, MS, Brazil. 

Of the 37 candidates presenting CMIA non-negative results for Chagas disease, 3 showed reagent results (absorbance > 1.2), and 34 showed inconclusive results (absorbance between 0.8 and 1.2) during serological screening at the blood banks. The CMIA test for screening for Chagas disease was standardized and distributed to blood banks by the Brazilian Ministry of Health. 

For the positive control group, 18 individuals with confirmed Chagas disease infections were included from the Chagas Disease Study Group at the University Hospital of Campinas State University (GEDoCh/Unicamp), all with positive serology and epidemiology for Chagas disease. For the negative control group, there were 11 health participants, all with negative serology and epidemiology for Chagas disease (i.e., with no infections).

All participants included were adults aged more than 18 years old. Each participant was invited to participate in this study, and upon acceptance, they signed an appropriate informed consent form and answered the questionnaire, and blood samples were collected for serological and molecular tests. The Ethics Committee approved this study via numbers CAAE: 80618017.9.0000.5404 from Campinas State University (Unicamp) and CAAE: 82928518.1.3001.5404 from Mato Grosso do Sul Federal University (UFMS).

### 2.2. ELISA to Detect Anti-T. cruzi Antibodies

The ELISA test (ABCAM Anti-Chagas IgG Human ELISA Kit, catalog ab178637) was performed on all serum samples from the blood donation candidates (*n* = 35), and samples considered a positive control (*n* = 18) and negative control (*n* = 11). All patients were evaluated by this test to obtain a standard result. 

To perform the test, about 2 mL of serum was submitted to the ELISA test to detect immunoglobulin G, according to the protocol of the commercial Anti-Chagas IgG Human ELISA Kit. Samples were analyzed in duplicate, and the value was obtained from the average of the two results, obtained with a spectrophotometer. 

In each assay, the positive, negative, white, and cutoff controls were applied. The sample’s absorbance was measured at 450 nanometers in a Beckman DU800 (Beckman Instruments, Brea, CA, USA) spectrophotometer. The cutoff value was measured in each assay, and toward this value, the samples that showed an absorbance value > 10% of the cutoff were said to be positive. Samples showing an absorbance value < 10% of the cutoff were said to be negative, and samples showing absorbance values equal to the cutoff were said to be inconclusive.

### 2.3. IFAT to Detect Anti-Leishmania Antibodies

The reactions were performed following the protocol from the *Biomanguinhos* Institute kit, manufactured by the Osvaldo Cruz Foundation, Rio de Janeiro, Brazil, with the IFAT kit for Human Leishmaniasis, batch 189LH001Z. In each assay, 35 samples from the blood donation candidates were tested, including 18 positive controls for CD and 11 negative controls, and samples showing titration ≥ 1:80 were said to be positive.

### 2.4. DNA Extraction

DNA extraction was performed on 66 samples from whole blood (donation candidates, positive control, and negative control). Red cell lysis was performed using approximately 6 mL of blood. The tubes were centrifuged at 752 g for 12 min. The plasma was removed, and the pellet was transferred to the conic tube. The red cell lysis buffer (NH_4_Cl + NH_4_HCO_3_) was used to complete the 15 mL of the mixture and then centrifuged as described above. This step was repeated two times. The next step consisted of washing the mixture with the TKM1 buffer (Tris-HCl 2M + KCl 1M + MgCl_2_ + EDTA 0.2 + H_2_O) and Triton. The mixture was centrifuged as described above, and the supernatant was discarded, generating a leucocyte pellet. The pellet was submitted to DNA extraction using the High Pure PCR Template Preparation (Roche, Mannheim, Germany) commercial kit, code number 11796828001, following the manufacturer’s protocols. DNA was eluted in 200 uL of an elution buffer provided in the kit and stocked at −20 °C.

Additionally, the absence of inhibitors and DNA quality were analyzed by amplifying the human β-globin gene.

### 2.5. Nested PCR to Detect T. cruzi Satellite DNA

DNA samples of the 35 subjects from the blood donation candidates were tested, including 18 positive controls for CD and 11 negative controls. These DNA samples were subjected to amplification of the parasite satellite DNA using the sequences TCZ1 (CGAGCTCTTGCCCACACGGGTGCT) and TCZ2 (CCTCCAAGCAGCGGATAGTTCAGG) and, subsequently, in the Nested PCR, the sequences TCZ3 (TGCTGCA(G/C)TCGGCTGATCGTTTTCGA) and TCZ4 (CA(A/G)G(C/G)TTGTTTGGTGTCCAGTGTTGTGA). In the first step, the reactions comprised 1.0 µL of DNA, KCl 50 mM; Tris-HCl 10 mM pH 8.4; MgCl2 2.5 mM; dNTPs 200 mM; 0.1 mM of each primer (TCZ1 and TCZ2); and 2 U of Taq DNA polymerase for a final volume of 20 μL. In the second step, the reaction comprised the same reagents, and the DNA was replaced by product from the first reaction. In the first reaction, the samples passed through 30 cycles of denaturation at 94 °C for 1 min, annealing at 60 °C for 1 min, and extension at 72 °C for 1 min and 30 s. The 25 subsequent cycles were denaturation at 94 °C for 1 min, annealing at 65 °C for 1 min, and extension at 72 °C for 1 min and 30 s. The second reaction passed through 25 cycles of denaturation at 94 °C for 40 s, annealing at 55 °C for 40 s, and extension at 72 °C for 1 min [34,35,36]. The amplification was analyzed in agarose gel 2% stained with ethidium bromide and seen under UV light. DNA from a Chagas disease patient was used as a positive control.

### 2.6. PCR to Detect Leishmania Kinetoplast DNA

In 66 samples (donation candidates, positive controls, and negative controls), *Leishmania* genus detection was performed via amplification of a segment of 120 bases pairs of kDNA parasite using the primers A 5′-(G/C) (G/C(C/G)CC(A/C)CTAT(A/T)TTACACCAACCCC and B: 5′-GGGGAGGGGCGTT CTGCGAA). The reaction was prepared using the Go Taq Green Master Mix (Promega, Fitchburg, WI, USA), one μM of each primer, one μL of DNA, and water to complete 20 μL. Subsequently, the reactions were submitted to denaturation at 94 °C for 4 min, 35 cycles of 94 °C for 30 s, 60 °C for 30 s, and 72 °C for 30 s, and a final extension at 72 °C for 10 min [37,38,39]. The amplification was analyzed in agarose gel 2% stained with ethidium bromide and seen under UV light. DNA from *L. infantum*, *L. braziliensis*, and *L. amazonensis* were used as positive controls. 

### 2.7. Real-Time PCR for DNA Quantification and Melting Curve Analysis (MCA)

The standard curve was built from cultured *T. cruzi* obtained from an infected individual and previously genotyped as DTU II in our laboratory. For the *Leishmania* spp. standard curve, the *Leishmania (Leishmania) infantum chagasi* strain MHOM/BR/1972/LD was provided by the “Department of Animal Biology, Campinas State University (UNICAMP), Campinas”. For both cases, the parasites were counted in a Neubauer chamber, and the number of parasites obtained was 1.87 × 10^8^ parasite equivalent/mL for *T. cruzi* and 7.8 × 10^7^ par. eq/mL for *L. infantum.* Subsequently, two samples (6 mL each) of the blood of a non-infected person were spiked with the initial parasite’s amount and submitted to DNA extraction as described above. Using different dilutions of human blood spiked with parasites, we constructed the standard curve for both targets, ranging from 1.87 × 10^1^ for *T. cruzi* to 7.8 × 10^0^ for *L. infantum*. These serial dilutions were used as the standards for sample quantifications in the qPCR.

The qPCR reactions performed on 37 samples for both targets were composed of the Power SYBR™ Green PCR Master Mix (ThermoFisher, catalog number 4367659, Texas, USA), 0.2 mM of each primer, 1.5 µL of DNA, and water to achieve a final volume of 20 µL. The primer sequences for *T. cruzi* satellite DNA were Cruzi 1: 5′ASTCGGCTGATCGTTTTCGA3′ and Cruzi 2: 5′ 5′AATTCCTCCAAGCAGCGGATA3′ [40]. The reaction cycle was 95 °C for 10 min (1x), 95 °C for 15 s, 58 °C for 30 s, and 72 °C for 30 s, repeated 40x and posteriorly 95 °C for 15 s and 58 °C for 30 s (1x). For the amplification of the *L. infantum* kDNA, the primers (0.2 mM) were MLF: 5′-CGTTCTGCGAAAACCGAAA-3′ and reverse MLR: 5′-CGGCCCTATTTTACACCAACC-3′ [41,42]. The reaction cycle was 95°C for 10 min (1x), 95 °C for 30 s, 60 °C for 20 s, and 72 °C for 20 s, repeated 45x. For both *T. cruzi* and *L. infantum*, the temperature for the melting curve analysis was from 25 °C to 90 °C, increasing by 1 °C and calculated automatically by the real-time Rotor-Gene 6000 (Corbett LifeScience, California, USA) equipment. Samples were analyzed in duplicate in each reaction. Three replicates of one of the described standard-curve DNAs were used as amplification controls. No template control (NTC) samples were included in each reaction, indicating the absence of contamination in the reactions. 

The standard curve was built using different concentrations of DNA samples of parasites *T. cruzi* and *L. infantum* [40,41,42]. To estimate the cutoff value, the reaction containing different concentrations of parasite DNA was repeated five times. The limit of detection of the method was the lowest concentration of samples that showed at least 95% amplification.

For the MCA, the qPCR data for both targets were collected over seven different days and analyzed through descriptive measures, e.g., mean, standard deviation, minimum, median, maximum, and variation coefficient. The Mann–Whitney test was used to compare the melting temperature between both groups (*T. cruzi* and *L. infantum*). 

## 3. Results

A total of 66 participants were enrolled in this study. Clustering the subjects, the blood donor candidates (*n* = 37) had a mean age of 44.4 ± 13.4 years, with 20 males (54.1%) and 17 females (45.9%). The State of origin of these participants were as follows: 18 from São Paulo (48.65%), 6 from Mato Grosso do Sul (16.22%), 3 from Paraná (8.11%), 3 from Bahia (8.11%), 2 from Minas Gerais (5.41%), and 1 from each of the following States, equivalent to 2.7%: Ceará, Rio de Janeiro, Alagoas, Pernambuco, and Rio Grande do Sul (Table 1).

In the positive control group *(n* = 18), the mean age was 62.8 ± 9.9, with 6 of them being male (36.4%) and 12 female (66.7%). The origins of the subjects were as follows: eight from Minas Gerais (44.4%), four from São Paulo (22.2%), two from Paraná (11%), and one from each of the following States, equivalent to 5.6%: Bahia, Alagoas, Ceará, and Paraíba (Table 1).

The negative control group (*n* = 11) had a mean age of 40.3 ± 13.3, four of them being male (36.4%) and seven female (63.6%). The origin of the subjects was São Paulo State (Table 1).

Most blood donor candidates had completed secondary school (40.54%), followed by high school (37.84%), while 13.5% had completed primary school, and 8.11% had not completed primary school. Among the positive controls, 83.4% had completed primary school. In the negative control group, 72.7% had completed high school (Table 1). 

Of the 37 samples of blood from donor candidates, 35 were evaluated using ELISA, and in nine of them, 24.3% (9/35) showed a positive result. Two samples were concordant from ELISA and CMIA (Table 2). In the positive control group, 17 of 18 samples (94.4%) showed positive ELISA results, and all 11 samples (100%) from the negative control group showed negative results from ELISA (Table 3). 

Among the blood donor candidates (*n* = 37), two samples (HCPS26, HMS2) were excluded from the ELISA test due to missing samples. Two samples (HCPS26, HMS8) were excluded from qPCR and nPCR due to sample issues. The nPCR was able to detect 12 positive results for CD (34.28%), 18 (51.43%) were negative, and 5 (14.28%) were inconclusive when the reaction was repeated three times. In the positive control group, 7 of the 18 samples (38.8%) were positive from nPCR. In the negative control group (*n* = 11), all the samples showed negative results (Table 3).

**Figure 1:**
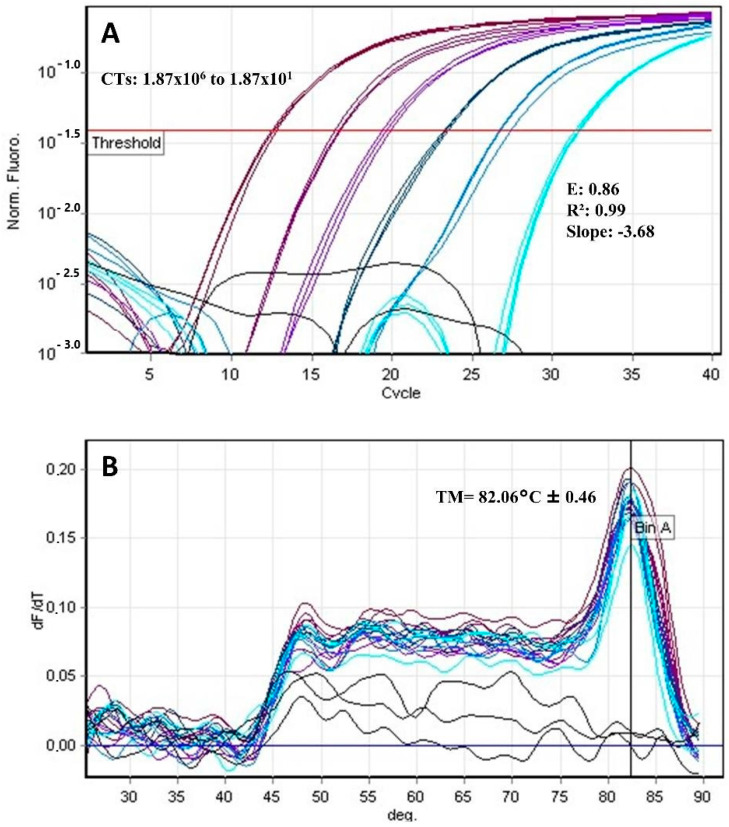
qPCR SYBR Green. (**A**) CT: Cycle threshold in the *T. cruzi* standard curve shows serial dilutions of 1.87 × 10^6^ to 1.87 × 10^1^; Efficiency (E) = 0.86, R^2^ = 0.99, and Slope = −3.68. (**B**) *T. cruzi* melting curve analysis with MT = 82.06 °C ± 0.46.

**Figure 2:**
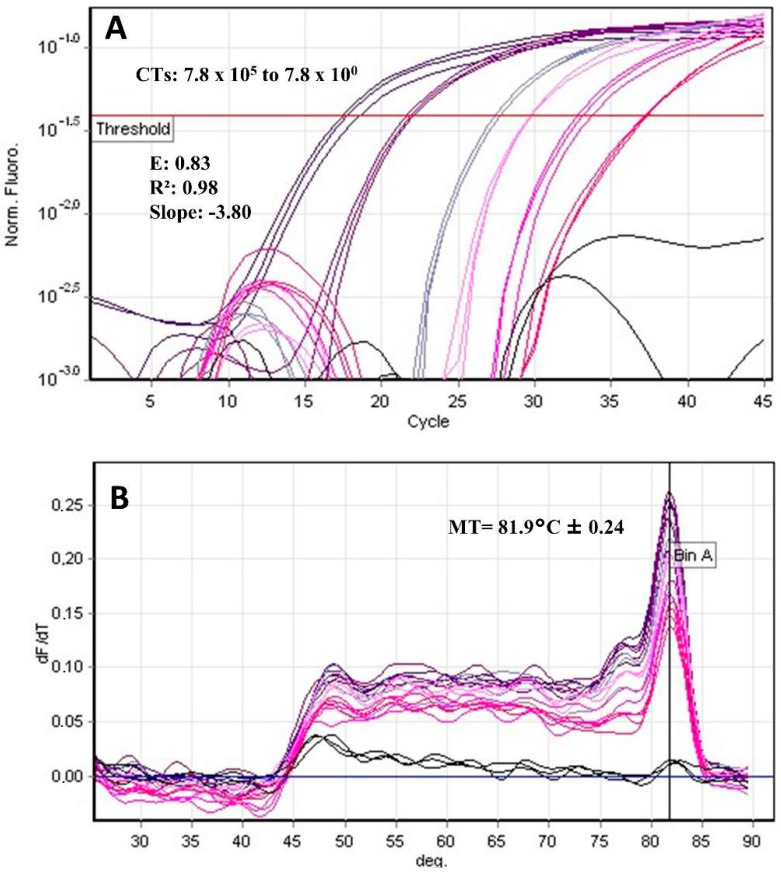
qPCR SYBR Green. (**A**) CT: Cycle threshold in the *L. infantum* standard curve shows serial dilutions of 7.8 × 10^5^ to 7.8 × 10^0^; Efficiency (E) = 0.83, R^2^ = 0.98, and Slope = −3.80. (**B**) *L. infantum* melting curve analysis with MT = 81.9 °C ± 0.24.

For qPCR, using different dilutions of human blood spiked with parasites, the analyses showed E = 0.86 (0.9–1.1), r² = 0.99 (> 0.99), slope = −3.68, and a melting temperature of 82.06°C ± 0.46 for the *T. cruzi* standard curve and E = 0.83, r² = 0.98, slope= −3.80, and a melting temperature of 81.9 °C ± 0.24 for the *L. infantum* standard curve (Figure 1 and Figure 2). In the repeatability tests, we observed a heterogeneous coefficient of variation among the different parasite concentrations for both targets. For MCA, the comparison was evaluated using the Mann–Whitney test and showed a significant value at *p* < 0.0001. However, the differentiation between *T. cruzi* and *Leishmania infantum* cannot be considered due to temperature overlap (Table 4).

As a diagnosis test, the qPCR for *T. cruzi* was considered quantifiable in the samples that showed a value ≥ 0.002 par Eq/mL. Thus, of the 35 samples, 9 were quantified, and 2 were considered detectable but not quantifiable. Then, 11 (31,42%) were considered positive for Chagas disease from qPCR. Five of the eighteen samples (27.7%) from the positive control group were positive from qPCR, and the eleven samples from the negative control group were undetectable (Table 3). 

For tests performed for Chagas disease diagnostics for non-negative serology, donors that showed two positive serologies (CMIA and ELISA) or one positive molecular test (nPCR and/or qPCR) (Table 2) were taken as being positive. Both the nPCR and qPCR methods agreed on 20 cases (57.14%) and were discordant in 10 cases (28.57%). The other five cases could not be compared because the nPCR was inconclusive in the repeatability tests. When making comparisons to the positive control group in this study, the sensitivity of nPCR was 62%, and that of qPCR was 58%, while the specificity for both methods was 100%. 

In summary, of the 37 samples evaluated using the CMIA, ELISA, nPCR, and qPCR tests, 18 (48.6%) were positive for Chagas disease. Of the three positive CMIAs (HCPS 14, HCPS 16, and HCPS 27), HCPS 16 and HCPS 27 were confirmed as positives. Of the 34 blood donor candidates with non-negative serology, 16 were confirmed as positive (Table 2). All patients were forwarded to reference centers to perform complementary and clinical exams. 

For leishmaniasis, of the 35 samples with non-negative serology for CD and tested by IFAT, only 1 sample (2.85%) was positive (1:80). This blood donor candidate (HCPS 04) showed inconclusive CMIA for Chagas disease, ELISA negative, nPCR negative, and qPCR positive for CD (0.07 par Eq/mL). All other 34 samples were negative for IFAT. All samples from positive and negative control groups for Chagas disease were also negative for IFAT. 

The PCR for *Leishmania* was performed in 36 samples of blood from donation candidates, and all were negative, as qPCR for *Leishmania* showed 37 negative results of the 37 samples analyzed. The samples of positive controls (*n* = 18) and negative controls (*n* = 11) also presented negative results for leishmaniasis.

## 4. Discussion

In Brazil, recent data from the *Agência Nacional de Vigilância Sanitária* [1,2] showed that 0.15% of all donated blood bags were blocked due to the presence of anti-*T. cruzi* antibodies. Due to the high sensitivity of the tests applied for screening in the blood banks, it is possible to consider cross-reaction with leishmaniasis caused by the genetic similarity among the parasites and also the existence of co-endemic areas that receive migratory flows from the endemic areas. However, there is a lack of data for leishmaniasis, because serological screening is not mandatory in the laboratory screening of blood donations. Data from studies show anti-*Leishmania* antibodies detected by different tests in some areas of the Brazil, among other countries [6,7,8,9,37,39].

For identifying *Leishmania* spp., one sample (HCPS 04) was positive from IFAT at a 1:80 dilution; however, this sample was also positive for Chagas disease from qPCR. One possible explanation for this finding, which has been corroborated by previous studies, is the antigenic similarities between these agents, and cross-reactions in the available detection tests [19,20]. In this study, the serological IFAT test was used to detect leishmaniasis, available from the Brazilian Ministry of Health, given to public reference laboratories, due to its reproducibility. The sensitivity and specificity of the kit are variable, ranging from 75.4% to 92% when compared to the direct agglutination test, ELISA, and rapid test rk39. IFAT and ELISA showed more common cross-reactions with *T. cruzi* [39]. 

The sensitivity of the serological tests depends on the antigens used. Currently, ELISA tests are more sensitive and specific, since they are produced with recombinant antigens of specific epitopes that decrease the chances of cross-reactions with other trypanosomatids [21,22,23]. For Chagas disease, the Anti-Chagas IgG Human ELISA Kit (Abcam) was used, with a present sensitivity of 100% and a specificity of 98.9%, according to the manufacturer’s manual. When testing the known positive samples for Chagas disease (positive controls), 17 of the 18 samples were positive, so the sensitivity of the ELISA test was 94.5%. Since the *T. cruzi* population is polyclonal, possibly having more than one DTU in the host, the test may not have been able to identify the antibodies produced. This sample GCP15 with a negative ELISA result was positive in nPCR, showing the existence of a circulating parasite or parasite fragments. The specificity of this test was 100% because all samples from the negative control group showed negative results. 

The CMIA test for Chagas disease is distributed by the Brazilian Ministry of Health to all blood banks, with standardized protocols. Therefore, the negative and positive controls of this study were not evaluated by this test. Our objective was not to evaluate the CMIA. The inconclusive or positive CMIA results obtained at blood banks were the starting point for testing the samples with other serological and molecular methodologies to try to exclude cases of cross-reactions between *T. cruzi* and *Leishmania* spp. Since the CMIA cutoff in blood banks is high, there is no point in testing CMIA-negative samples, since they are considered to be true negatives. The hypothesis is that the samples with doubtful or positive CMIA may show this result given probable cross-reactivity.

The nPCR test detected 12 (34.28%) positive results for Chagas disease, 18 (51.43%) were negative, and 5 (14.29%) were inconclusive in the three replicated PCRs. The qPCR was positive in eleven cases (31.42%), detecting a mean of 0.06 par Eq/mL (0.002–0.15). Both methods agreed on 20 cases (57.14%) and were discordant in 10 cases (28.57%). The molecular biology diagnostic methods are highly specific and have relative sensitivity. Compared to the positive control group, in this study, the nPCR and qPCR sensitivities were 62% and 58%, respectively, while the specificity was 100%. The low and intermittent parasite load in the chronic cases of Chagas disease may justify this result [43]. 

We observed low concordance between nPCR and qPCR in DNA samples extracted from the blood of donor candidates. This issue has been discussed in other works that have addressed the molecular diagnosis of *Trypanosoma cruzi* [24,44]. For nPCR, the samples were previously tested with an internal control, the human β-globin gene. Only the samples that presented amplification for β-globin, which indicates the absence of reaction inhibitors and the quality of DNA extraction, were taken to PCR for the pathogen. All techniques were performed in triplicate. Furthermore, in each reaction, every care was taken to avoid cross-contamination, with the inclusion of negative controls and tubes with reagents only, without DNA. The probable explanation for the low concordance is nPCR or qPCR inhibition, due to amplicon excess in the second nPCR reaction, interfering with the sensitivity of the tests, or due to the quenching of the fluorescence or by hindering a dye from a binding DNA. Other probable inhibitors are hemoglobin, hematin, or immunoglobulin G (IgG) present in samples extracted from blood [45,46]. This does not preclude the performance of PCRs as an alternative or additional technique for clarifying inconclusive cases of Chagas disease. In addition to the objective of clarifying these cases at blood banks, it is important for donor candidates to know what led to the blockage of their blood bag, and if they have a positive result for Chagas disease, whereafter they should have the opportunity to receive a follow-up and be treated by the specialized and multidisciplinary medical service offered by the SUS (Unified Health System) in Brazil.

Considering the molecular methods, nine of the positive samples from qPCR and nine positive samples from nPCR had negative ELISAs for DC, reinforcing the possibility of false-negative serological results, which may occur due to the immunological status of the patient or the absence of the production of antibodies to certain strains of parasites [44]. Additionally, there are some cases of CD detection through other methods associated with the clinical or epidemiological status of the patient, which showed persistent inconclusive serology [34,35,36]. The qPCR and PCR for leishmaniasis showed 100% negative results, reinforcing the specificity of molecular techniques. 

The melting temperature shows the temperature at which the amplicons begin to dissociate. To analyze the melting temperature, the qPCR is performed using SYBR Green fluorophore, which allows for the analysis of only one molecular target per reaction. Thus, to distinguish the genera *Trypanosoma* and *Leishmania* in one reaction, one would need to design primers to indicate the parasite genera. This was the major limitation of this study, and it could be explored in future studies.

Here, the melting temperatures of the MCA for *T. cruzi* and *L. infantum* were statistically significant; however, some values overlapped. Thus, more tests using different targets to possibly distinguish the parasites through melting temperature are needed, including tests of different qPCR reaction conditions. Previous studies have shown that six different *T. cruzi* DTUs can be successfully identified using high-resolution melting (HRM). However, to identify the *Leishmania* species, the available data are highly variable. In many cases, the method could differentiate the subgenus *L. (Leishmania)* from subgenus *L. (Viannia)* or allocate the parasites to different groups according to the interval of the melting temperature. The SYBR Green methodology does not allow for multiplex reactions, because it is DNA-intercalating, i.e., it can bind to any DNA strand. By contrast, the Taqman methodology allows for multiplex reactions but does not generate a melting curve, which is the main analysis of the study [30,31,32,33].

Although the qPCR has been shown to be a promising method for improving the diagnosis of Chagas disease and leishmaniasis, there is a lack of method standardizations for both diseases. Previous research conducted in international studies has identified the most reasonable target for the molecular detection of Chagas disease. The results showed the best four methods, three of which are directed to satellite DNA and one to the kDNA of *T. cruzi*, for PCR hot start and qPCR [24]. No standardizations have been published for the identification or quantification of the *Leishmania* species. For both parasites, the issues related to the difficulty of the standardization method involve the biological sample used, the amount of the biological sample collected, the molecular target, the DNA extraction technique, and the variability of the parasite in different areas [47].

CD and leishmaniasis are considered neglected tropical diseases that are still challenging for the scientific community and for patients. This study did not show the presence of *Leishmania* DNA in the blood of donation candidates, but it was possible to demonstrate that serological tests may cross-react, as was already reported in other studies [22,48]. The inclusion of leishmaniasis serology in blood banks may help control disease transmission, especially in endemic areas. 

The data presented here show the importance of performing two different tests for Chagas disease screening at blood banks, as recommended by the WHO [3]. In the case of the persistency of inconclusive tests, or discordance between tests, molecular tests should be used for confirmation. Although the PCR for *T. cruzi* DNA identification showed relative sensitivity, its specificity is high and can achieve 100%. All blood banks in Brazil already perform molecular tests for other diseases such as viral hepatitis, HIV, and malaria [48,49], so laboratories already have equipment and specialized staff that can perform conventional or quantitative molecular diagnostics for both CD and leishmaniasis in endemic areas. This strategy can lead to increased transfusion safety and better characterizations for the results of inconclusive serological laboratory screenings or screenings with inconsistent results.

## Figures and Tables

**Table 1:** Sociodemographic and epidemiological characteristics of the study population (*n* = 66).

Variable	Donation Candidates (*n* = 37)	Positive Controls (*n* = 18)	Negative Controls (*n* = 11)
**Gender**			
male	20 (54.1%)	06 (33.3%)	04 (36.4%)
female	17 (45.9%)	12 (66.7%)	07 (63.6%)
**Age Mean ± SD (years)**	44.4 ± 13.4	62.8 ± 9.9	40.3 ± 13.3
**Birth location (State)**			
São Paulo	18 (48.65%)	04 (22.2%)	11 (100%)
Mato Grosso do Sul	06 (16.22 %)	-	-
Bahia	03 (8.11%)	01 (5.6%)	-
Paraná	03 (8.11%)	02 (11 %)	-
Minas Gerais	02 (5.41%)	08 (44.4%)	-
Ceará	01 (2.7%)	01 (5.6%)	-
Rio de Janeiro	01 (2.7%)	-	-
Alagoas	01 (2.7%)	01 (5.6%)	-
Pernambuco	01 (2.7%)	-	-
Rio Grande do Sul	01 (2.7%)	-	-
Paraíba	00 (0.00%)	01 (5.6%)	-
**Educational level**			
None	03 (8.11%)	-	-
Primary School	05 (13.51%)	15 (83.4%)	01 (9.1%)
Secondary School	15 (40.54%)	02 (11%)	02 (18.2%)
Higher Education	14 (37.84%)	01 (5.6%)	07 (72.7%)

**Table 2:** Results and interpretation of tests for Chagas disease.

		Chagas Disease Tests				
		CMIA Blood Banks	ELISA	nPCR	qPCR	Interpretation Tests
**Blood donor candidates**	HCPS 1	I	NR	P	ND	Positive
	HCPS 2	I	NR	N	0.132 Par Eq/mL	Positive
	HCPS 3	I	NR	P	0.007 Par Eq/mL	Positive
	HCPS 4	I	NR	N	0.07 Par Eq/mL	Positive
	HCPS 5	I	NR	P	0.005 Par Eq/mL	Positive
	HCPS 6	I	NR	P	0.004 Par Eq/mL	Positive
	HCPS 7	I	NR	N	ND	Inconclusive
	HCPS 8	I	NR	N	ND	Inconclusive
	HCPS 9	I	NR	IRT	0.1 Par Eq/mL	Positive
	HCPS 10	I	R	IRT	ND	Inconclusive
	HCPS 11	I	NR	N	0.002 Par Eq/mL	Positive
	HCPS 12	I	NR	N	ND	Inconclusive
	HCPS 13	I	NR	P	0.07 Par Eq/mL	Positive
	HCPS 14	R	NR	N	ND	Inconclusive
	HCPS 15	I	NR	P	ND	Positive
	HCPS 16	R	R	N	ND	Positive
	HCPS 17	I	NR	N	ND	Inconclusive
	HCPS 18	I	NR	P	D < 0.002 Par Eq/mL	Positive
	HCPS 19	I	R	P	ND	Positive
	HCPS 20	I	NR	N	ND	Inconclusive
	HCPS 21	I	NR	P	ND	Positive
	HCPS 22	I	R	N	ND	Inconclusive
	HCPS 23	I	NR	N	ND	Inconclusive
	HCPS 24	I	R	N	ND	Inconclusive
	HCPS 25	I	NR	WS	WS	Inconclusive
	HCPS 26	I	WS	P	ND	Positive
	HCPS 27	R	R	P	D < 0.002 Par Eq/mL	Positive
	HCPS 28	I	R	N	ND	Inconclusive
	HMS 1	I	NR	IRT	ND	Inconclusive
	HMS 2	I	WS	N	ND	Inconclusive
	HMS 3	I	NR	N	ND	Inconclusive
	HMS 4	I	NR	N	ND	Inconclusive
	HMS 5	I	NR	P	ND	Positive
	HMS 6	I	R	IRT	ND	Inconclusive
	HMS 7	I	NR	IRT	ND	Inconclusive
	HMS 8	I	NR	WS	WS	Inconclusive
	HMS 9	I	R	N	0.15 Par Eq/mL	Positive
		***n* = 37** **03 P/34 I**	***n* = 35** **09 R/26 NR**	***n* = 35** **12 P/18 N/05 IRT**	***n* = 35** **11 D/24 ND**	***n* = 37** **18 P/19 I**

CMIA: chemiluminescent microparticle immunoassays; ELISA: enzyme-linked immunosorbent assay; nPCR: nested PCR; qPCR: quantitative PCR; CD: Chagas disease; WS: without sample; I: inconclusive—for CMIA value (OD 0.8 to 1.2); IRT: inconclusive repeatability test in PCR; HCPS: Hemocentro de Campinas or Hematology and Hemotherapy Center; HMS: Hemocentro de Mato Grosso do Sul or Hemosul; I: inconclusive; NR: non-reagent; R: reagent. Interpretation: Positive: two positive serologies or one molecular positive test; Inconclusive: one serology inconclusive and/or one no reagent and/or inconclusive repeatability test in PCR. For qPCR: ≥0.002 par Eq/mL—quantifiable; D—detectable, not quantifiable < 0.002 par Eq/mL.; ND—not detectable.

**Table 3:** Results for Chagas disease.

Study Population/Positive Tests for Chagas Disease	CMIA	ELISA	nPCR	qPCR
Non-negative for CD (*n* = 37)	37	09/35 (24.3%)	12/35 (34.28%)	11/35 (31.42%)
Positive controls (*n* = 18)	NT	17/18 (94.4%)	7/18 (38.8%)	5/18 (27.7%)
Negative controls (*n* = 11)	NT	0/11 (0%)	0/11 (0%)	0/11 (0%)

CMIA: chemiluminescent microparticle immunoassays; ELISA: enzyme-linked immunosorbent assay; nPCR: nested PCR; qPCR: quantitative PCR; CD: Chagas disease; NT: not tested.

**Table 4:** Repeatability tests for the Melting Temperature (MT) of the *Leishmania infantum* and *Trypanosoma cruzi* by qPCR SYBR Green.

Parasite	Variable	Samples Tested	Average Temperature °C	Standard Deviation	Minimum Temperature °C	Median Temperature °C	Maximum Temperature °C	Variation Coefficient	*p*-Value
*Leishmania infantum*	MT	179	**81.28**	**0.58**	79.20	81.30	83.00	0.72	**<0.0001**
	CT	113	31.25	7.94	12.45	33.22	44.53	25.42	
*Trypanosoma cruzi*	MT	200	**81.86**	**0.45**	80.20	81.80	83.50	0.55	
	CT	186	25.72	7.14	12.14	26.94	40.00	27.87	

## Data Availability

Data available on request due to restrictions eg privacy or ethical.
